# Biochemical and molecular mechanisms of probiotic action: current concepts and emerging therapeutic applications

**DOI:** 10.3389/fnut.2026.1964473

**Published:** 2026-10-07

**Authors:** Alexey A. Churin, Lyudmila O. Sokolyanskaya, Anastasia P. Lukina, Olga V. Karnachuk

**Affiliations:** 1 Laboratory of Microbial Technologies, Tomsk State University, Tomsk, Russia; 2 Department of Plant Physiology, Biotechnology, and Bioinformatics, Tomsk State University, Tomsk, Russia

**Keywords:** engineered probiotics, gut barrier function, immune modulation, microbial metabolites, next-generation probiotics, pharmacomicrobiomics, probiotics, short-chain fatty acids

## Abstract

Probiotic effects are mediated by a network of biochemical transformations and molecular signaling events that influence host physiology through local and systemic mechanisms. This review examines how probiotic microorganisms generate functional metabolites, including short-chain fatty acids, bile acid derivatives, and indole compounds, and how these molecules interact with host receptors and intracellular signaling pathways such as NF-κB, JAK/STAT, FXR, TGR5, AhR, and inflammasome-mediated signaling. In addition to metabolite-mediated signaling, probiotics may inhibit pathogens through organic acid production, bacteriocins, and quorum-quenching mechanisms, while also contributing to epithelial barrier integrity through modulation of tight-junction proteins and mucosal defenses. These interconnected pathways affect inflammation, metabolic regulation, host–microbe communication, and host defense against pathogens. The review further discusses pharmacomicrobiomic interactions, engineered probiotics, and emerging delivery technologies, highlighting the importance of strain specificity, host context, and microbiome composition in determining therapeutic outcomes. These findings underscore the importance of mechanistic frameworks for the rational development of targeted microbiome-based interventions.

## 1 Introduction

The scientific understanding of probiotics has advanced considerably from its empirical origins. Once viewed mainly as general dietary supplements, probiotics are increasingly investigated as strain-specific, mechanism-informed interventions with potential relevance to disease prevention and management (1). Their effects are best considered in relation to the microbial functions, host responses, and ecological context in which they operate rather than as a universal property shared by all probiotic microorganisms.

Probiotic formulations have traditionally been dominated by lactobacilli and bifidobacteria. The former broad genus *Lactobacillus* has undergone a major phylogeny-based reclassification; consequently, many well-known probiotic species are now assigned to genera such as *Lactiplantibacillus*, *Lacticaseibacillus*, and *Limosilactobacillus*. For example, *Lactobacillus plantarum*, *Lactobacillus rhamnosus*, *Lactobacillus casei*, *Lactobacillus paracasei*, *Lactobacillus reuteri*, and *Lactobacillus fermentum* are currently recognized as *Lactiplantibacillus plantarum*, *Lacticaseibacillus rhamnosus*, *Lacticaseibacillus casei*, *Lacticaseibacillus paracasei*, *Limosilactobacillus reuteri*, and *Limosilactobacillus fermentum*, respectively; in contrast, *Lactobacillus acidophilus* and *Lactobacillus gasseri* remain within the emended genus *Lactobacillus* (2).

The expanding microbiome-therapeutics landscape also requires clear distinctions among related terms. Conventional probiotics are live microorganisms that, when administered in adequate amounts, confer a health benefit on the host (3). Next-generation probiotics (NGPs) are rationally selected or functionally characterized commensal strains with disease-relevant mechanisms and potential therapeutic applications, but they are not inherently pharmaceutical products (4, 5). By contrast, live biotherapeutic products (LBPs) are defined live microorganisms developed as therapeutic products under pharmaceutical-style development and regulatory frameworks (6). NGPs and LBPs are related but non-interchangeable categories: an NGP may be developed as an LBP when advanced as a defined therapeutic product within an appropriate regulatory framework. Engineered probiotics are genetically modified microbial systems designed to sense specified environmental signals or deliver defined therapeutic payloads and may serve as candidates for LBP development (7). Postbiotics are preparations of inanimate microorganisms and/or their components that confer a health benefit on the host (8).

Beyond their established role in supporting intestinal microbial homeostasis, probiotics, postbiotics, and related microbiome-based interventions are increasingly being investigated as potential modulators of host–microbiota interactions in inflammation-associated and cancer-related settings. Recent reviews suggest that probiotic-based approaches may influence dysbiosis, epithelial barrier function, microbial metabolites, inflammatory signaling, and tumor-associated host–microbe interactions; however, their therapeutic value in colorectal cancer remains an emerging area that requires further clinical validation (9). In colorectal cancer-related experimental models, a polyherbal and probiotic-based immunostimulatory formulation has shown immunomodulatory, antimicrobial, and antiproliferative activity, supporting the potential value of combining beneficial microorganisms with bioactive phytochemicals in dysbiosis-associated disease settings (10). In addition, metabolite-enriched preparations derived from healthy microbiota or probiotic cultures have been associated with reduced *Fusobacterium* growth, adhesion, and invasion, together with changes in colorectal cancer cell viability, migration, apoptosis, and inflammatory signaling (11). These findings should be interpreted as early mechanistic and preclinical evidence rather than established therapeutic efficacy; nevertheless, they illustrate how microbiome-based interventions may be developed as adjunctive, mechanism-informed strategies for dysbiosis-associated disease (9).

This review advances the argument that the biological and translational relevance of probiotics is best understood through an integrated systems-level framework rather than through isolated pathway- or disease-specific descriptions. It is organized around a nine-block mechanistic structure that connects microbial gene functions and products with host phenotypes across metabolic regulation, receptor signaling, epithelial barrier biology, immune modulation, antimicrobial defense, pharmacomicrobiomics, engineered therapeutics, and delivery strategies. In this framework, microbiota-derived metabolites may influence host physiology through interconnected immunometabolic and barrier-associated processes, while host–microbiota interactions can also have consequences beyond the intestine (12–14). By linking these levels of analysis, the review examines how mechanistic understanding may help explain variable probiotic responses and inform the development of more targeted microbiome-based interventions, while recognizing that many proposed applications remain translationally unresolved (15).

## 2 SCFA and energy metabolism

The gut microbiota plays a central role in systemic energy metabolism and colonic homeostasis by fermenting non-digestible carbohydrates into short-chain fatty acids (SCFAs), principally acetate, propionate, and butyrate (13, 16, 17). Particular structural features of prebiotic carbohydrates, such as the *β*(2 → 1) glycosidic linkages in fructo-oligosaccharides, make them resistant to human digestive enzymes and allow them to reach the colon intact for selective use by beneficial microbes. Under low-oxygen conditions in the intestinal lumen, probiotic strains typically rely on anaerobic glycolysis, contributing to the formation of lactate and pyruvate (16). Through homolactic and heterolactic fermentation pathways, these intermediates are subsequently converted into a broader SCFA pool (17). These microbially derived SCFAs serve as major energy substrates for colonocytes and have also been reported to act as postbiotic signaling molecules with potential roles in host immune and metabolic regulation (18). The principal biochemical and molecular mechanisms through which probiotics influence host physiology are summarized in Figure 1, providing an integrated framework for the mechanistic pathways discussed throughout this review. A taxonomic overview of the beneficial and clinically relevant bacterial groups discussed in this review, including their functional characteristics and therapeutic relevance, is provided in Supplementary Table S1.

**Figure 1:**
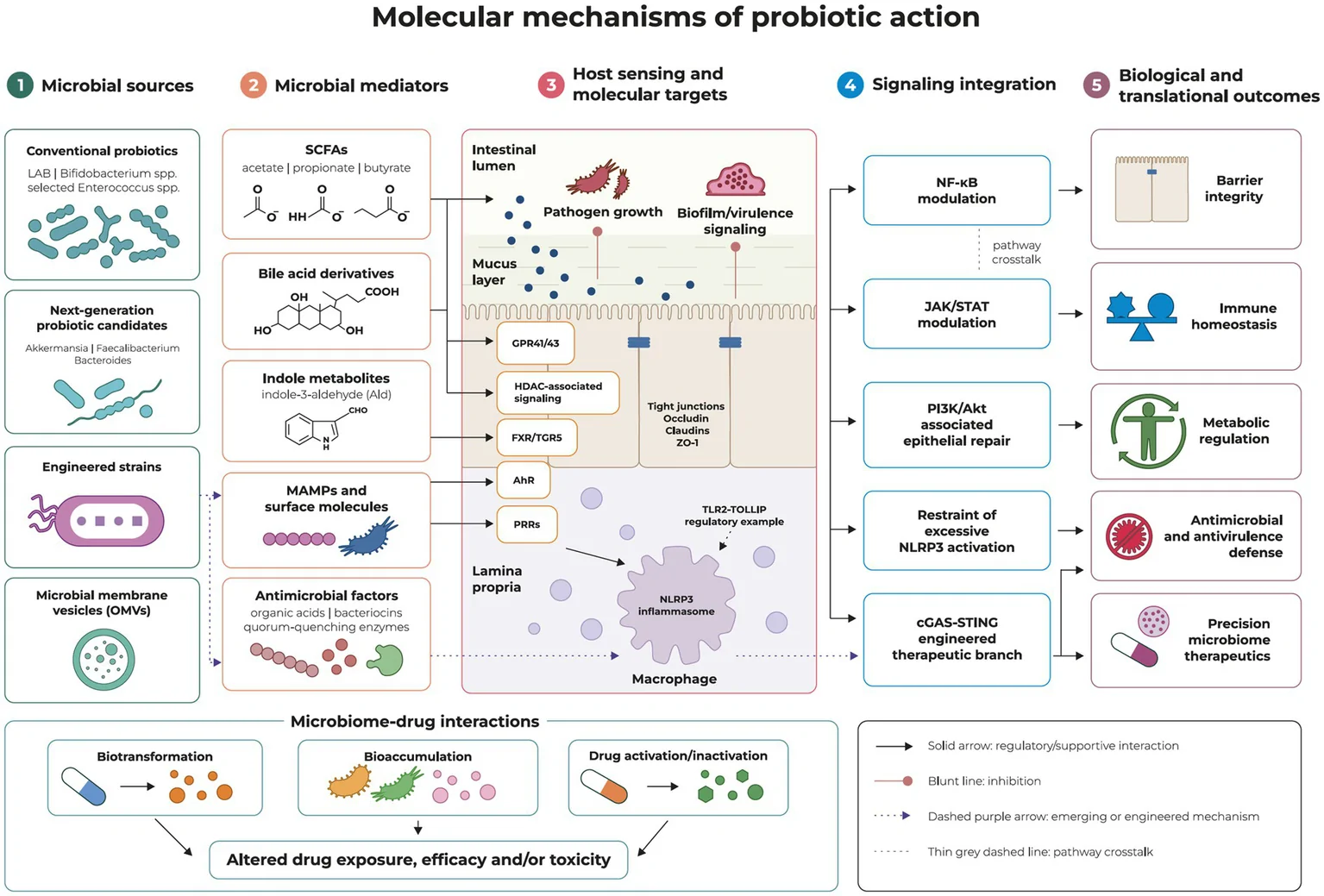
Molecular mechanisms of probiotic action. Schematic overview of the principal biochemical and molecular mechanisms through which probiotic microorganisms and their derivatives may influence host physiology. The figure is organized into five hierarchical levels: (1) microbial sources, including conventional probiotics, next-generation probiotic candidates, engineered strains, and microbial membrane vesicles (OMVs); (2) microbial mediators, including short-chain fatty acids, bile acid derivatives, indole metabolites, microbe-associated molecular patterns (MAMPs), surface molecules, and antimicrobial factors; (3) host sensing and molecular targets at the intestinal interface, including pattern-recognition receptors, G-protein-coupled receptors, nuclear receptors, and tight-junction complexes; (4) signaling integration, including NF-κB, JAK/STAT, PI3K/Akt, the NLRP3 inflammasome, cGAS–STING, and related pathways; and (5) biological and translational outcomes. The outcomes layer groups four biological endpoints (barrier integrity, immune homeostasis, metabolic regulation, and antimicrobial and antivirulence defense), one pharmacomicrobiomic endpoint (altered drug exposure, efficacy and/or toxicity), and one translational endpoint (precision microbiome therapeutics). OMVs are shown as non-living delivery platforms that may transport microbial cargos to host cells without requiring durable live colonization. Dashed purple arrows indicate engineered therapeutic branches, whereas thin gray dashed lines indicate pathway crosstalk. Solid arrows represent regulatory or supportive interactions, and blunt-ended lines indicate inhibition.

Available clinical studies and meta-analyses suggest that SCFA-modulating prebiotic and probiotic interventions may improve selected metabolic outcomes, including obesity-related dysfunctions, although effect sizes remain variable across formulations, populations, and study designs (17, 19). Administration of a human-origin probiotic cocktail containing *Lactobacillus* and *Enterococcus* species has been reported to increase SCFA production, particularly butyric acid, while also modifying the local colonic environment (20). Mechanistically, *in vivo* studies have shown that commensal microbe-derived butyrate promotes the differentiation of colonic Foxp3 + regulatory T cells, thereby suppressing mucosal inflammation (21, 22). In experimental models, lactate and other probiotic-derived factors have been associated with Wnt/β-catenin signaling involved in epithelial restitution. SCFAs have also been reported to support intestinal barrier function through effects on tight-junction regulation, including increased or stabilized expression of proteins such as ZO-1 and occludin in some models (23–25).

Understanding of microbial energy metabolism has changed substantially over the past two decades. Earlier work largely relied on high-performance liquid chromatography to quantify luminal acetate, propionate, and butyrate, but these measurements provided limited insight into intracellular targets. A major conceptual shift came with *in vitro* T-cell polarization assays and genetic knockout models, which demonstrated that SCFAs actively shape host immune circuitry rather than serving merely as passive energy substrates (21). Recent reviews suggest that the structural characteristics of prebiotic carbohydrates, including chain length and glycosidic linkage patterns, may contribute to differences in microbial fermentation and downstream metabolic responses, although the extent of these effects likely depends on the specific substrate, dose, and host context (25, 26).

Taken together, SCFAs illustrate how a microbial metabolic product may link substrate availability, epithelial physiology, and immune regulation. Their concentrations and biological effects are shaped by diet, antibiotic exposure, and the composition and functional capacity of the resident microbiome (19). Table 1 summarizes the principal biochemical mechanisms considered throughout this review, including metabolite production, antimicrobial activity, barrier support, immune modulation, and microbiome-drug interactions. The next section considers bile acids, another major class of microbial metabolites whose host effects likewise depend on microbial transformation and receptor context.

**Table 1:** Principal biochemical mechanisms of probiotic action.

Mechanistic domain	Key microbial products or processes	Primary host targets	Main biological effects	Representative examples and key references
Short-chain fatty acid production	Fermentation of non-digestible carbohydrates; acetate, propionate, butyrate	Colonocytes, epithelial cells, T cells, G-protein-coupled receptors	Energy supply, immune regulation, barrier support	*Bifidobacterium, Lactobacillus*, butyrate-producing consortia (16–18, 21)
Bile acid biotransformation	Bile salt hydrolase, deconjugation, secondary bile acids	FXR, TGR5, enteroendocrine cells, liver	Metabolic regulation, GLP-1 secretion, lipid homeostasis	BSH-active probiotic strains (27–30)
Amino acid and tryptophan metabolism	Indoles, kynurenine-related metabolites	AhR, immune cells, neural pathways	Mucosal immune balance, neuroimmune signaling	Psychobiotic strains, indole-producing taxa (25, 34, 35, 42)
Direct antimicrobial activity	Organic acids, bacteriocins, antimicrobial peptides	Pathogenic bacteria, microbial membranes	Pathogen inhibition, reduced colonization	Lactic acid, pediocin, MccJ25 (67–69)
Quorum quenching and antivirulence effects	Lactonases, acylases, signal degradation	Bacterial communication systems	Biofilm attenuation, virulence suppression	AiiA-like lactonases and related quorum-quenching enzymes (70–72)
Barrier reinforcement	Modulation of tight-junction-associated pathways, mucus support	Epithelial cells, tight junction proteins	Reduced permeability, mucosal protection	Occludin, ZO-1, Muc2 (52, 84–86)
Immune receptor signaling modulation	MAMP recognition, strain-specific immunomodulators	TLRs, NLRs, NF-κB-related pathways	Reduced inflammation, immune homeostasis	TLR2/TLR6-linked signaling, NF-κB and inflammasome modulation (48, 51, 54, 57)
Microbiome–drug interaction pathways	Drug biotransformation, bioaccumulation, enzyme-mediated activation/inactivation	Drug metabolism systems, liver, intestinal epithelium	Altered efficacy and toxicity	Irinotecan, digoxin, sulfasalazine (29, 106, 122)

This table summarizes the principal mechanistic domains through which probiotics may influence host physiology. Representative examples and selected references are provided in the final column.

## 3 Bile acid and lipid metabolism

The regulation of host lipid and systemic energy metabolism by the gut microbiota depends in large part on the enzymatic biotransformation of bile acids. Once considered primarily digestive surfactants, bile acids are now recognized as endocrine and immunomodulatory signaling molecules. The bacterial enzyme bile salt hydrolase catalyzes the deconjugation of conjugated bile acids in the gut (27). Following this step, unabsorbed bile acids undergo further microbial transformations, including dehydroxylation and epimerization, generating a diverse pool of secondary bile acids. These microbially derived metabolites may influence systemic metabolic processes through interactions with host receptors, particularly the farnesoid X receptor (FXR) and the G-protein-coupled bile acid receptor TGR5 (28–30). Accumulating evidence suggests that selected probiotic strains may modulate bile acid metabolism and related FXR/TGR5 signaling pathways, with potential effects on hepatic lipid metabolism, glucose homeostasis, and intestinal inflammation via the gut–liver axis (31–34).

Available reviews and meta-analyses suggest that probiotics and synbiotics may offer modest, formulation-dependent benefits in metabolic syndrome, obesity, and type 2 diabetes, although the magnitude of effect varies across populations and outcomes (19, 32). Meta-analyses also indicate modest improvements in selected markers of metabolic dysfunction-associated steatotic liver disease, including liver enzymes, lipid parameters, and inflammatory indices (19). At a mechanistic level, secondary bile acids have been reported to activate TGR5 on enteroendocrine L-cells and to stimulate glucagon-like peptide-1 (GLP-1) secretion, providing a plausible link between microbial bile acid metabolism, glucose homeostasis, and appetite regulation (25). In the context of cardiovascular risk, strains with high bile salt hydrolase (BSH) activity have been associated with reductions in serum cholesterol and LDL-C, plausibly through decreased bile acid reabsorption and increased hepatic cholesterol utilization for bile acid synthesis (27). More broadly, altered bile acid profiles have been associated with cognitive dysfunction in neurodegenerative disease, including Alzheimer’s disease, supporting continued investigation of a microbiota–gut–brain–bile axis (25).

Work on microbial bile acid metabolism has expanded markedly over the past three decades. Earlier studies were largely focused on bile salt hydrolase activity as a bacterial survival mechanism against bile toxicity and, secondarily, as a potential contributor to luminal cholesterol lowering (33). The discovery of host bile acid receptors such as FXR and TGR5 fundamentally transformed this framework by establishing bile acids as systemic signaling molecules rather than simple detergents (29). More recently, shotgun metagenomics combined with untargeted LC–MS metabolomics has improved the resolution with which the intestinal bile acid landscape and microbe-associated influences on host receptor signaling can be characterized (15). These approaches may support more informed prediction of metabolite–receptor interactions, although their value for therapeutic selection remains to be established in prospective clinical studies.

Collectively, bile acid–microbiota interactions represent a biologically plausible route through which microbial functions may influence metabolic and inflammatory phenotypes. Their clinical relevance remains uncertain because bile acid profiles, microbial enzyme activity, diet, and host metabolic state vary substantially between individuals. Future studies should therefore distinguish receptor-level mechanistic observations from reproducible clinical effects and identify the conditions under which modulation of bile acid pathways is most likely to be informative (15, 19, 31).

## 4 Amino acid metabolism and the gut-brain Axis

The regulation of host neuroimmune responses by the gut microbiota is influenced in part by microbial amino acid metabolism, linking intestinal microbial activity to central nervous system signaling. Among the most important pathways in this gut–brain communication is microbiota–host tryptophan metabolism (34, 35). Tryptophan can be metabolized through the kynurenine pathway, initiated by host indoleamine 2,3-dioxygenase, generating downstream metabolites that have been associated with either neuroprotective effects, such as kynurenic acid, or neurotoxic effects, such as quinolinic acid (25, 35). In parallel, specific probiotic strains may influence serotonin-related pathways relevant to sleep, circadian regulation, and broader neurobehavioral homeostasis (35). Microbial tryptophan catabolism can also generate indole-3-aldehyde, an AhR ligand implicated in mucosal immune homeostasis. Available evidence therefore suggests that probiotics may affect mood-related outcomes through intersecting immune, neuroendocrine, and neurotransmitter-associated mechanisms, although their relative contribution in humans remains uncertain (31, 34).

Recent reviews and preclinical studies suggest that psychobiotic strains and their postbiotic products may have relevance to depression, anxiety, and related disorders, but the clinical evidence remains heterogeneous (36, 37). Experimental studies have also associated modulation of the microbiota–gut–nerve axis with improvement in neuropathic pain, whereas human psychobiotic studies more commonly report variable mood- and stress-related outcomes (38–40). Related metabolomic evidence suggests that dysbiosis may shift tryptophan metabolism toward a more neurotoxic kynurenine profile, marked by quinolinic acid predominance over kynurenic acid, whereas targeted probiotic modulation may help restore a less inflammatory and potentially more neuroprotective balance (25, 41–43).

Research on the gut–brain axis has broadened from an early emphasis on vagal and neuroendocrine communication to the use of metagenomics and metabolomics for characterizing neuroactive microbial metabolites (44, 45). Studies of IDO1- and AhR-related signaling have provided mechanistic support for a role of microbial metabolites in neuroimmune regulation, while computational approaches are beginning to assist the interpretation of metabolite–receptor relationships (15, 42, 46, 47).

Overall, the amino acid–microbiome–gut–brain axis remains a promising area for adjunctive investigation in mood disorders, inflammatory bowel disease, and painful diabetic neuropathy. However, clinically reproducible benefit is limited by variation in baseline microbiota, intervention design, and outcome assessment, as well as uncertainty about the relevance of circulating or luminal metabolites to central nervous system effects. Longitudinal human studies that link microbial metabolic profiles with predefined clinical and neuroimmune endpoints are needed to clarify these relationships (15, 45).

## 5 Immune receptor Signaling and inflammatory cascades

The regulation of host inflammatory responses by the gut microbiota is strongly shaped by interactions between microorganism-associated molecular patterns (MAMPs) and host pattern-recognition receptors, particularly Toll-like receptors and Nod-like receptors. Table 2 outlines the major signaling pathways and molecular targets discussed in this section, including NF-κB, JAK/STAT, TLR2/TLR6, NLRP3 inflammasome, FXR, TGR5, AhR, PI3K/Akt, cGAS–STING, and TOLLIP-mediated regulation. Recognition of MAMPs by these receptors can initiate inflammatory cascades that include NF-κB activation (48–50). Among the intracellular pathways involved, NF-κB remains one of the best characterized regulators of innate and adaptive immune responses (51). Upon receptor activation, adaptor molecules such as MyD88 initiate a kinase cascade involving IRAKs and the IκB kinase complex, thereby promoting IκB degradation and NF-κB nuclear translocation, with subsequent induction of chemokines, adhesion molecules, and pro-inflammatory cytokines (51, 52). In parallel, intracellular sensors such as the NLRP3 inflammasome assemble in response to danger signals and mediate the maturation of IL-1β and IL-18 through caspase-1 activation (53). Probiotics may counter excessive inflammatory activation through strain-specific regulatory mechanisms, including modulation of TLR2/TLR6-linked signaling and support of epithelial barrier integrity (52). TOLLIP is an important negative regulator of TLR-mediated inflammatory signaling and may help restrain downstream NF-κB activation (54, 55). These pathways provide a plausible mechanistic basis for investigating probiotic interventions as adjunctive approaches in immune-mediated disorders; however, their clinical relevance is likely to vary by strain, host context, and disease phenotype (56).

**Table 2:** Major signaling pathways and molecular targets modulated by probiotics.

Pathway or receptor	Upstream trigger	Downstream signaling event	Representative biological effect	Representative examples and key references
NF-κB	TLR stimulation, inflammatory cues, microbial ligands	IκB degradation, NF-κB nuclear translocation, induction of pro-inflammatory cytokines	Attenuation of inflammatory signaling and cytokine production	NF-κB pathway modulation in inflammatory models (51, 57, 65)
JAK/STAT	Cytokine signaling and inflammatory stimulation	STAT phosphorylation and transcriptional regulation of immune-response genes	Dampening of inflammatory cascades in bowel disease models	JAK/STAT-associated probiotic immunomodulation (57, 66)
TLR2/TLR6	Probiotic-associated molecular patterns and bacterial surface structures	MyD88-linked epithelial signaling and downstream immune regulation	Barrier support, immune modulation, and tightening of epithelial junctions	TLR2/TLR6-dependent probiotic signaling (50, 52, 64)
NLRP3 inflammasome	Danger-associated signals, epithelial stress, inflammatory activation	Caspase-1 activation and maturation of IL-1β and IL-18	Restraint of inflammasome-related inflammation	Inflammasome-linked regulation in probiotic-relevant inflammatory settings (51, 54)
FXR	Secondary bile acids generated by microbial metabolism	Regulation of metabolic and bile-acid-responsive gene networks	Modulation of lipid metabolism, intestinal homeostasis, and glucose-related signaling	Microbial bile acid–FXR signaling (28, 29, 123)
TGR5	Microbially modified bile acids	Enteroendocrine activation and GLP-1-associated signaling	Effects on glucose homeostasis, endocrine regulation, and metabolic signaling	Bile acid–TGR5 signaling in metabolic regulation (25, 29, 30)
AhR	Indole derivatives and other tryptophan metabolites of microbial origin	Transcriptional control of mucosal immunity, barrier responses, and neuroimmune signaling	Promotion of immune tolerance and mucosal homeostasis	AhR-linked effects of microbial tryptophan metabolism (34, 35, 42)
PI3K/Akt	Strain-specific epithelial receptor activation, including TLR-associated signaling	Cell survival, epithelial restitution, and barrier-related signaling	Support of epithelial repair and barrier maintenance	PI3K/Akt involvement in probiotic epithelial signaling (52, 64)
cGAS-STING	Engineered microbial systems, microbial nucleic acid sensing, tumor-targeted therapeutic constructs	Innate immune activation and downstream antitumor signaling	Programmable immunomodulation and therapeutic targeting in engineered probiotic platforms	STING-directed engineered probiotic strategies (91, 92, 97, 99)
TOLLIP-mediated regulation	TLR activation with compensatory negative-feedback signaling	Suppression or restraint of excessive downstream inflammatory activation	Limitation of cytokine amplification and inflammatory overactivation	TOLLIP-associated negative regulation of TLR signaling (52, 54, 55)

This table outlines the principal host receptors and intracellular signaling pathways discussed in this review, together with their upstream triggers, downstream signaling events, and observed probiotic-associated effects. Representative examples and selected references are provided in the final column.

Accumulating *in vitro*, animal, and clinical evidence supports the immunomodulatory capacity of probiotics in inflammatory disorders. In intestinal epithelial models, probiotic cocktails containing *Lactobacillus* and *Bifidobacterium* species have been shown to attenuate NF-κB-associated signaling by downregulating key pathway intermediates, including TIRAP, IRAK4, and NEMO, with concomitant reductions in IL-6 and IL-1β production (57). These effects are consistent with negative feedback mechanisms in TLR signaling, in which TOLLIP contributes to restraint of excessive activation (51, 54, 55). Although such models provide mechanistic support, clinical findings in ulcerative colitis and other inflammatory disorders remain strain- and outcome-dependent (55, 56). Beyond NF-κB, JAK/STAT has also emerged as an immunoregulatory target, with probiotic interventions reported to modulate inflammatory signaling in bowel disease models (57).

Experimental and selected clinical studies have also explored probiotic-associated effects on epithelial restitution, wound healing, and inflammatory tissue repair, although the strength of evidence differs substantially across settings (58, 59). In DSS-induced colitis models, *Leuconostoc lactis* EJ-1 and *Escherichia coli* M-17 have been associated with reduced inflammatory activity and attenuated colitis severity through NF-κB-related and macrophage-polarization mechanisms (60–62). Probiotic and probiotic-like strategies have also been investigated in chemotherapy-induced intestinal mucositis, where NF-κB-driven inflammatory amplification may contribute to epithelial injury (63).

Advances in receptor biology, phosphorylation-state analysis, and protein-localization studies have made it possible to examine how defined microbial effectors influence epithelial and immune signaling. These studies support a model in which different probiotic features may converge on TLR2-dependent pathways, with downstream effects involving NF-κB, PI3K/Akt, and epithelial barrier maintenance (52, 57, 64, 65).

Translation of immune-signaling findings into consistent clinical benefit remains challenging. Responses may depend on the administered strain, host genetics, baseline microbiome composition, and local inflammatory context; in disorders such as Crohn’s disease, variation in innate immune pathways may add a further source of heterogeneity. Future studies should test whether defined microbial or postbiotic interventions produce reproducible effects on prespecified immune and barrier-related biomarkers before broader clinical claims are made (51, 66).

## 6 Direct antimicrobial mechanisms and enzymatic actions

The direct antagonism of intestinal pathogens by probiotic bacteria involves secreted antimicrobial compounds and enzymes that interfere with microbial signalling. Organic acids, particularly lactic acid, can contribute to the antimicrobial milieu and may permeabilize Gram-negative bacteria by disrupting the outer membrane (67, 68). Probiotic strains may also produce bacteriocins, including peptides such as pediocin that disrupt membrane integrity. More specialized molecules, such as the lasso bacteriocin MccJ25, can enter susceptible bacteria through specific receptors and inhibit transcription by blocking the RNA polymerase secondary channel (69). Beyond direct growth inhibition, probiotics and their products may interfere with pathogen communication through quorum quenching. Lactonases and acylases can disrupt acyl-homoserine lactone signalling and thereby attenuate quorum-dependent virulence-associated behaviours (70–72). Cell-envelope proteinases from lactic acid bacteria also hydrolyze dietary proteins into smaller bioactive peptides that may contribute to host defense and other health-related effects (73).

Evidence for quorum quenching remains predominantly experimental. In bacterial pathogen models, the *Bacillus*-derived lactonase AiiA reduces acyl-homoserine lactone accumulation and attenuates virulence in organisms such as *Erwinia carotovora* and *Pseudomonas aeruginosa* (72). Pathogens may also develop adaptive responses to antimicrobial peptides; for example, *Enterococcus faecalis* has been reported to alter membrane fluidity and increase positively charged phospholipids, reducing susceptibility to cationic peptides (74). By comparison, selected probiotic strategies have been evaluated clinically for acute gastrointestinal infections, antibiotic-associated diarrhea, and *Clostridioides difficile* infection, although effects vary among formulations and patient groups (75–80).

Methodological progress has moved the field from activity-based assays, such as agar diffusion tests, toward molecular and structural characterization of bacteriocins and quorum-quenching enzymes. High-throughput screening and structural approaches now support identification of candidate quorum-sensing inhibitors and analysis of their potential modes of action (69, 70).

Quorum-sensing pathways are being explored as antivirulence targets because they regulate biofilm formation, toxin production, and coordinated pathogenic behaviour without necessarily compromising bacterial viability (81). This rationale does not preclude resistance: experimental evolution studies have identified mechanisms, including enhanced multidrug efflux in *P. aeruginosa*, that can reduce susceptibility to quorum-quenching compounds (82). Engineered probiotic vectors designed to deliver signal-disrupting activities within microbial communities remain an emerging concept rather than an established intervention (83). Their utility will depend on activity under physiological conditions, penetration of mature biofilms, and the evolutionary responses of target organisms.

## 7 Barrier function and epithelial integrity

The intestinal epithelium constitutes the primary physical and immunological interface between the host and the luminal environment. Probiotics may help maintain barrier integrity and immune homeostasis by acting across several defensive layers. The mucus layer secreted by goblet cells forms an outer barrier that overlies the epithelial glycocalyx and limits direct pathogen contact with the epithelium (84). Beneath the mucus layer, the paracellular space is sealed by apical tight junctions composed of transmembrane proteins such as occludin, claudins, and junctional adhesion molecules, which are linked to the actin cytoskeleton through scaffold proteins including zonula occludens-1 (ZO-1) (52, 85). Dysbiosis-associated disruption of intestinal permeability has been associated with altered immune activation and loss of mucosal homeostasis in autoimmune disease (53). Certain probiotic strains have been reported to influence the transcription, membrane localization, or functional stabilization of junctional proteins, potentially limiting paracellular exposure to luminal antigens, endotoxins, and pathogens (86).

Preclinical epithelial models provide support for strain-specific barrier effects. In Caco-2 monolayers, for example, *Bifidobacterium bifidum* BB1 selectively enhanced occludin expression and junctional localization without broadly altering other transmembrane tight-junction proteins (52). In animal models, probiotic administration has been associated with preservation of mucosal architecture, reduced pathological apoptosis, and maintenance of barrier-associated molecules such as ZO-1 and Muc2 (87). Clinical trials and meta-analyses likewise suggest that selected probiotic interventions may improve barrier-related outcomes and attenuate mucosal inflammation in disorders such as inflammatory bowel disease and antibiotic-associated diarrhea, although the magnitude and reproducibility of these effects remain context-dependent (75, 88).

The study of epithelial barrier regulation has progressed from functional *in vivo* permeability measurements to transwell-grown epithelial monolayers, which allow controlled assessment of barrier physiology through transepithelial electrical resistance and defined stimulation experiments (89). More recent studies have combined approaches such as siRNA silencing and confocal immunofluorescence to examine the localization and regulation of tight-junction proteins in response to defined probiotic stimuli (52).

Restoration of epithelial barrier function is therefore a relevant therapeutic consideration in disorders associated with increased intestinal permeability. Translation remains challenging because barrier responses are influenced by inflammatory tone, baseline microbiome composition, and strain-specific molecular activity. Future studies would benefit from pairing well-characterized formulations with measurable epithelial endpoints, including permeability assays and junctional-protein markers, rather than inferring barrier benefit from broad clinical outcomes alone (85, 86). An integrative overview of the major microbial metabolites and bioactive products discussed in this review, together with their principal host receptors, signaling pathways, and biological effects, is provided in Supplementary Table S2. The following section considers how these mechanistic insights are being extended through next-generation and engineered microbial platforms.

## 8 Next-generation probiotics and synthetic biology

Building on the terminology defined in the Introduction, recent work has expanded beyond conventional probiotic supplementation to include next-generation probiotics, live biotherapeutic products, and engineered microbial systems with more clearly specified functions (1). Synthetic biology and CRISPR-Cas-based approaches are being used to develop microbial chassis that may sense disease-relevant signals or deliver defined payloads at mucosal sites (90). *Escherichia coli* Nissle 1917 and *Lactococcus lactis*, for example, have been engineered to conditionally express anti-inflammatory mediators, with the aim of limiting local inflammatory signalling while reducing systemic exposure to conventional immunosuppressive agents (62, 88). Table 3 compares the main translational platforms discussed in this review, including conventional probiotics, synbiotics, postbiotics, NGPs, LBPs, engineered probiotics, encapsulated formulations, OMV-based systems, and pharmacomicrobiomic interventions.

**Table 3:** Translational applications of probiotics, postbiotics, and engineered live biotherapeutics.

Therapeutic platform	Core biological function	Potential application	Main advantage	Representative examples and key references
Conventional probiotics	Live microbial supplementation to support host–microbe homeostasis and functional resilience	Gastrointestinal disorders, metabolic support, adjunctive immune modulation	Broad familiarity, established use, relatively straightforward clinical translation	Classical probiotic formulations in human health and disease (31, 107, 121)
Synbiotics	Combined administration of probiotics and fermentable substrates to enhance selective microbial activity	SCFA enhancement, microbiota modulation, metabolic and mucosal support	Improved substrate availability and potential synergy between strain and substrate	Synbiotics as microbiome-modulating interventions (14, 19, 88)
Postbiotics	Use of microbial metabolites, inactivated cells, cell fragments, or secreted bioactive products	Immune modulation, barrier support, reduced-risk microbiome intervention	Greater stability, lower viability-related constraints, potentially improved safety in vulnerable populations	Postbiotic frameworks and emerging therapeutic relevance (18, 51, 124)
Next-generation probiotics	Rationally selected or functionally characterized strains with disease-relevant mechanisms	Precision microbiome intervention, inflammatory and metabolic disease targeting	Improved mechanistic specificity and stronger rationale for personalized use	Next-generation strains and microbiome-informed therapy concepts (1, 15, 31)
Live biotherapeutic products	Defined live microorganisms developed as therapeutic agents under pharmaceutical-style frameworks	Inflammatory disease, metabolic disorders, oncology, precision biologic intervention	Greater therapeutic specificity and translational alignment with drug-development pathways	LBP-oriented microbiome therapeutics (1, 102, 125)
Engineered probiotics	Synthetic biology-based microbial platforms designed to sense signals and deliver therapeutic payloads	Local anti-inflammatory therapy, tumor-targeted immunotherapy, programmable mucosal intervention	Tailored function, programmable activity, disease-specific targeting	Engineered *Escherichia coli Nissle* 1917, *Lactococcus lactis*, and related systems (90–92, 94)
Encapsulated formulations	Micro- or nanoencapsulation to protect microbes or co-delivered bioactives during gastrointestinal transit	Oral delivery optimization, site-specific release, improved survival and stability	Enhanced viability through gastric transit and improved delivery precision	Alginate-, pectin-, chitosan-, and protein-based delivery systems (14, 110, 126)
Outer membrane vesicle-based systems	Cell-free delivery of microbial bioactive cargo, including proteins, lipids, nucleic acids, and immune-active components	Postbiotic delivery, immune modulation, non-living alternatives to live engineered microbes	Functional delivery without durable colonization; attractive in higher-risk settings	OMV-based postbiotic and delivery concepts (90, 119)
Pharmacomicrobiomic interventions	Deliberate modulation of microbiome–drug interactions to affect drug metabolism, toxicity, or efficacy	Chemotherapy support, microbiome-informed pharmacotherapy, reduction of adverse drug effects	Expands probiotics into precision medicine and drug-response optimization	Microbiome–drug interaction strategies and adjunctive applications (63, 104, 106, 122)

This table summarizes the main therapeutic platforms currently shaping precision microbiome medicine, including conventional probiotics, next-generation probiotics, postbiotics, engineered live biotherapeutics, and advanced delivery strategies. Representative examples and selected references are provided in the final column.

Preclinical and early translational studies suggest that engineered microbial systems may be useful in selected disease settings. In murine colitis models, engineered *Bifidobacterium longum* strains have been associated with reduced experimental inflammation and host-protective responses (58). In oncology, the tumor-localized *Escherichia coli* Nissle 1917-based live biotherapeutic SYNB1891 was designed to deliver a STING agonist in the tumor microenvironment. It has been evaluated clinically, including in combination with the PD-L1 inhibitor atezolizumab, although its clinical role remains to be defined (91–95).

Related engineered approaches have also been explored in metabolic disease models. Modulation of cGAS–STING-related signaling has been associated with inflammatory, metabolic, and barrier-related outcomes in experimental non-alcoholic steatohepatitis, identifying this pathway as a potential therapeutic node rather than an established target (96–99). Several pathways summarized in Table 2, including cGAS–STING and TLR-linked signaling, are therefore being investigated as potential targets for engineered microbial platforms.

The development of engineered probiotics has progressed from proof-of-concept expression systems toward constructs that incorporate containment and control features. The conceptual evolution of probiotic therapeutics is illustrated in Figure 2, which links changes in mechanistic understanding and enabling technologies with potential clinical applications. Early studies, including IL-10-secreting *Lactococcus lactis* for experimental colitis, established feasibility but also drew attention to biosafety, environmental containment, and horizontal gene-transfer concerns (90). Current strategies include Cas9-assisted containment and genetic kill-switch or expiry circuits intended to limit replication and promote elimination after therapeutic activity (100–103).

**Figure 2:**
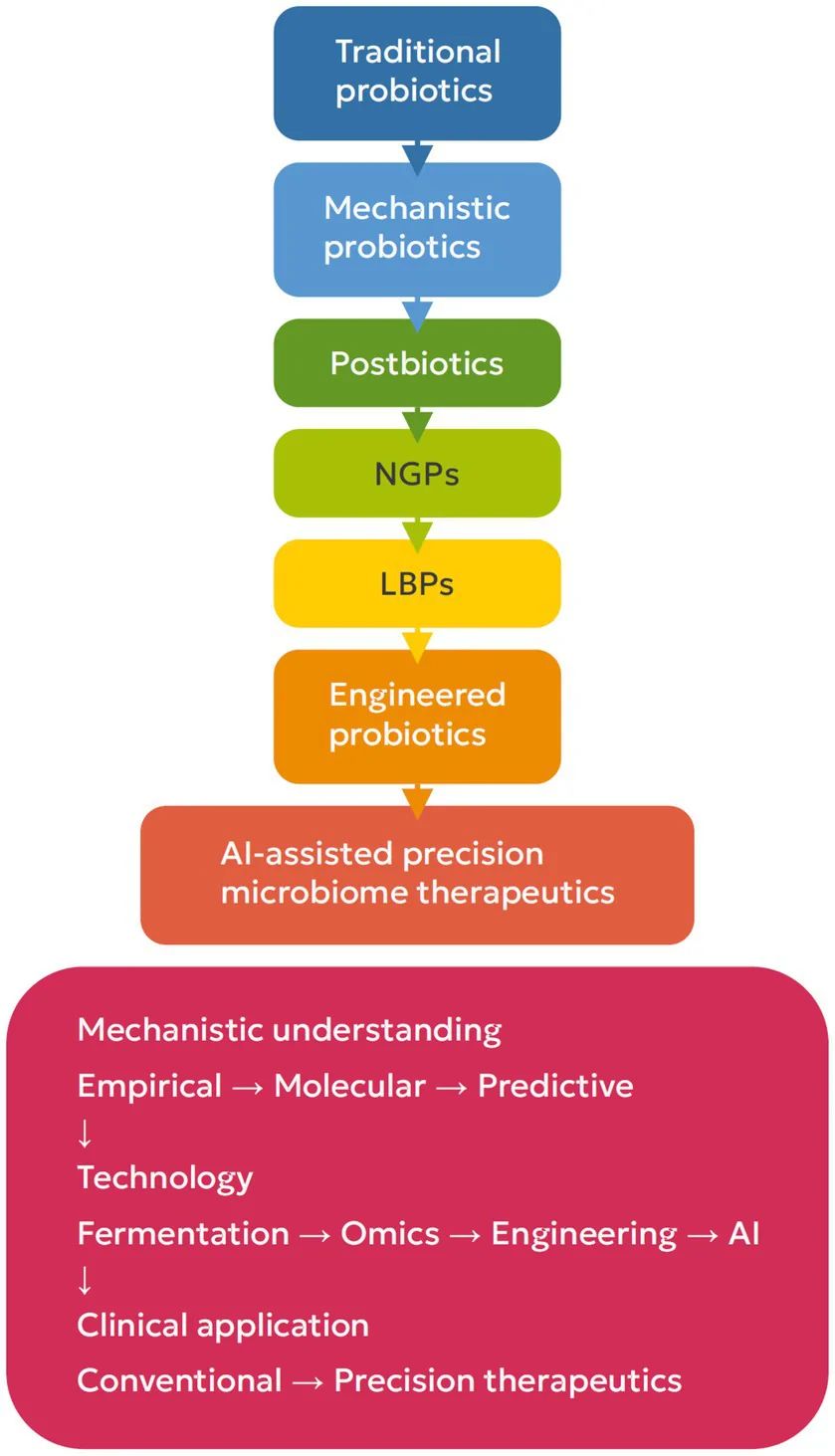
Evolution of probiotic therapeutics.

These developments indicate that NGPs, LBPs, and engineered microbes may eventually occupy distinct but complementary roles in microbiome-based therapeutics. Their implementation will depend on reliable engraftment or persistence where required, manufacturing consistency, biocontainment, regulatory oversight, and evidence from adequately designed human studies. Metagenomic, transcriptomic, and metabolomic data may help identify suitable microbial niches and guide chassis selection, but their routine use for patient stratification remains investigational (15, 94). The relevance of microbial function to therapeutic response becomes especially apparent in pharmacomicrobiomics, where resident microbes can alter the disposition and effects of administered drugs.

Conceptual overview illustrating the progression of probiotic research from traditional probiotics toward mechanistically defined precision microbiome therapeutics. The figure highlights the parallel evolution of biological understanding (from empirical observations to predictive mechanistic frameworks), enabling technologies (from fermentation to multi-omics, synthetic biology, and artificial intelligence), and clinical application (from conventional probiotic use toward precision therapeutics). Together, these advances have driven the transition from classical probiotics to postbiotics, next-generation probiotics (NGPs), live biotherapeutic products (LBPs), engineered probiotics, and AI-assisted precision microbiome therapeutics.

## 9 Pharmacomicrobiomics and microbiome–drug interactions

The intestinal microbiota can act as a metabolically active modifier of host responses to xenobiotics and may influence the pharmacokinetics and pharmacodynamics of orally administered drugs (104). These microbiome–drug interactions can be considered through three overlapping pathways:

1. *Direct microbial biotransformation*. Resident gut bacteria express enzymes that may chemically modify therapeutic molecules through reduction, hydrolysis, or deconjugation reactions in the intestinal lumen (29).
2. *Microbiome-mediated modulation of host drug metabolism*. Microbial metabolites, including SCFAs and bile acid derivatives, may influence host drug-metabolizing enzymes and transporters, including CYP3A4, CYP1A1, CYP2E1, and intestinal efflux transporters such as P-glycoprotein in the epithelium and liver (29).
3. *Microbial bioaccumulation*. Gut bacteria can sequester therapeutic molecules intracellularly without chemically modifying them, potentially reducing local drug availability and altering host drug exposure in the intestinal lumen (104, 105).

These processes also intersect with host pathways relevant to intestinal and systemic physiology. SCFAs may act through GPR41 and GPR43 in pathways associated with mucosal repair, whereas tryptophan-derived indoles can engage AhR and have been associated with reduced NF-κB-dependent inflammatory transcription and altered tight-junction regulation (13, 29). Microbially modified bile acids may likewise influence metabolic signalling through FXR and TGR5. Collectively, such interactions may affect local drug availability, intestinal absorption, systemic bioavailability, and overall drug exposure; their magnitude is likely to vary with the drug, microbial taxa and abundance, host physiology, and concurrent treatments (29).

Illustrative examples show how microbial activities can affect drug disposition and toxicity, although the strength of evidence differs across drugs and study systems.

- *Prodrug activation*. Sulfasalazine used in inflammatory bowel disease depends on intestinal bacterial azoreductases to cleave its azo bond and release 5-aminosalicylic acid in the colon. Germ-free animal models do not perform this conversion, supporting a microbial contribution to its activation (29, 104).
- *Drug inactivation*. *Eggerthella lenta* can inactivate digoxin through the glycoside-reducing cardiac glycoside reductase (*cgr*) operon, producing dihydrodigoxin and potentially contributing to variable drug response (29, 104).
- *Drug toxification and mucosal injury*. Host glucuronidation converts irinotecan to SN-38 glucuronide, which is excreted into the intestine; bacterial β-glucuronidases can regenerate the active metabolite SN-38 and have been implicated in treatment-associated intestinal toxicity. Adjunctive probiotic approaches, including *Lacticaseibacillus rhamnosus* GG and *Saccharomyces boulardii*, have been investigated to reduce chemotherapy-associated intestinal injury and diarrhea, but clinical results remain heterogeneous (63, 106).

Microbiome–drug interactions are bidirectional: antibiotics and other medications can alter microbial composition and functional capacity, thereby potentially changing subsequent drug metabolism, efficacy, or toxicity. Advances in metagenomics, metabolomics, stable-isotope tracing, and systematic *in vitro* screening have improved the ability to link particular transformations or bioaccumulation phenotypes to bacterial taxa and gene clusters, including the *cgr* operon of *E. lenta* (29, 104, 105, 107).

As summarized in Figure 3, this workflow combines experimentally defined microbial mechanisms of biotransformation, bioaccumulation, and enzyme-mediated activation or inactivation with metagenomic and metabolomic characterization. Computational stratification, including AI-assisted approaches, may help integrate these data and identify candidate microbiome-associated response patterns; however, this framework remains a developing research tool rather than a clinically standardized method for microbiome-informed pharmacotherapy (108).

**Figure 3:**
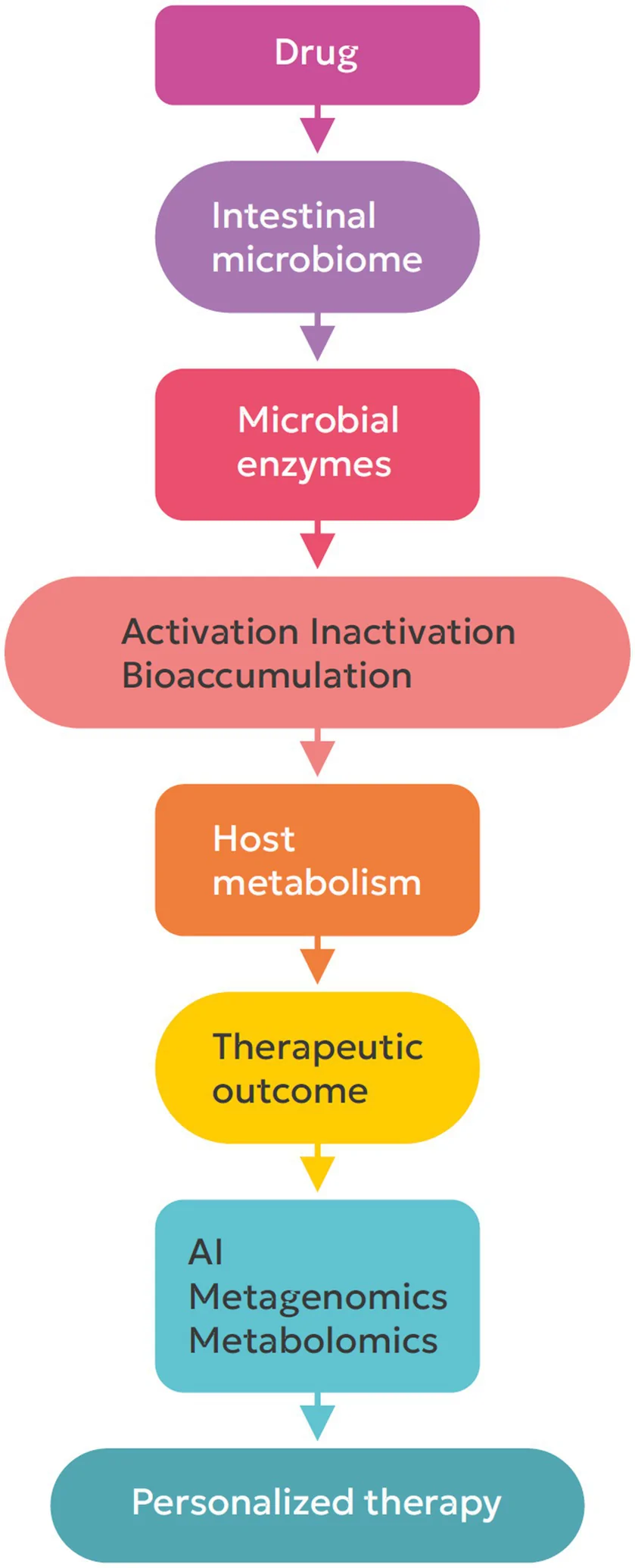
Conceptual overview illustrating how interactions between drugs and the intestinal microbiome influence therapeutic efficacy. Microbial enzymes may activate, inactivate, or bioaccumulate pharmaceuticals, thereby modifying host metabolism and clinical outcomes. The integration of metagenomics, metabolomics, and artificial intelligence provides opportunities to predict microbiome–drug interactions and support personalized therapeutic strategies.

At present, microbiome profiles may be useful for hypothesis generation and for identifying patient groups in whom altered drug response warrants further investigation. Their use for routine dose selection or monitoring, particularly for drugs with a narrow therapeutic index, remains limited by incomplete validation, temporal variability of the microbiome, and the absence of standardized clinical biomarkers.

Targeted synbiotic co-interventions may offer one route for exploring strain–drug interactions. For example, *Bifidobacterium animalis* B960 enhanced glibenclamide efficacy and reduced drug-associated adverse effects in a type 2 diabetes mouse model (109). Delivery technologies such as microencapsulation may also protect probiotic preparations during gastric transit and facilitate site-specific intestinal release (14). These examples should be interpreted as early translational concepts rather than established pharmacological strategies. The broader delivery systems and extra-intestinal consequences of microbiome-derived signals are considered in Section 9.

## 10 Advanced delivery systems and extra-intestinal axes

Recent developments in probiotic therapeutics extend beyond conventional oral supplementation and include engineered delivery systems as well as interventions directed at extra-intestinal host axes (14). Rather than representing a single technology class, these approaches range from protecting microbial viability during gastrointestinal transit to controlled local release, non-living vesicle delivery, tissue retention, and integration with other biotherapeutic modalities.

Micro- and nanoencapsulation provide a first level of control over gastrointestinal delivery. Biopolymer matrices, including alginate, pectin, chitosan, carboxymethyl cellulose, and whey proteins, have been used to protect probiotic cells from gastric acidity and to support release at intestinal pH (110). Single-cell coatings, layer-by-layer nanoencapsulation, and polysaccharide–protein hydrogel beads have been reported to improve survival of *Lactobacillus* and *Bifidobacterium* strains under simulated gastrointestinal conditions, in some studies by approximately 1–3 log units relative to non-encapsulated controls (110, 111). Beyond transit protection, biohybrid systems, including spore-forming bacterial platforms and nanoparticle-decorated microbial chassis such as *Escherichia coli* Nissle 1917 and selected *Bifidobacterium* strains, are being explored for targeted cargo delivery to inflamed mucosa or hypoxic tumor stroma (106, 111).

Non-living delivery platforms provide a complementary approach. Outer membrane vesicles (OMVs), including those released by Gram-negative commensals, are nanoscale vesicles that can interact with or traverse the intestinal mucus/glycocalyx interface. They may transport lipoproteins, lipopolysaccharides, nucleic acids, and effector proteins to host cells without requiring durable live bacterial colonization (90, 111). Engineered OMVs are being investigated for targeted delivery of antigens and nucleic-acid cargos, with early applications in mucosal immunomodulation and cancer therapy (90, 112, 113). Their potential advantages, including controlled cargo composition and avoidance of live-cell persistence, must still be balanced against unresolved issues of biodistribution, immunogenicity, and manufacturing standardization.

A separate direction aims to improve retention of therapeutic bacteria within defined tissue microenvironments. In a recent preclinical study, decorin-derived collagen-targeting molecular pili were chemically coupled to the surface of *Lactobacillus rhamnosus* (LR@MP), producing a surface display intended to promote adhesion to collagen-rich tumor extracellular matrix (114). In murine colorectal cancer and melanoma models, this approach was associated with approximately two-fold higher intratumoral bacterial accumulation, lower peripheral exposure, and localized antitumor activity; combination with PD-L1 blockade further enhanced the reported response (114). These findings are preliminary, and their relevance to human solid tumors will depend on safety, biodistribution, therapeutic window, and clinical feasibility.

Beyond gastrointestinal delivery, probiotic and postbiotic interventions are being investigated across several interconnected axes, including the gut–brain, gut–lung, gut–kidney, gut–nerve, gut–skin, and maternal–fetal axes (14, 115). The available evidence is uneven across these pathways and remains strongly dependent on strain, dose, model system, and host context. At the mucosal interface, cell-surface structures such as mucus-binding proteins, lipoteichoic acid, exopolysaccharides, pili, flagella, and surface-layer proteins may influence host–microbe interactions (115).

Along the gut–lung axis, microbial perturbation has been associated with altered pulmonary Th17/Treg balance, while probiotic or postbiotic interventions have shown immunomodulatory effects in experimental and selected clinical studies of allergic asthma and chronic obstructive pulmonary disease (116, 117). Along the gut–kidney axis, intestinal barrier dysfunction may contribute to systemic exposure to uremic solutes such as indoxyl sulfate and p-cresyl sulfate; synbiotic and probiotic approaches are being evaluated for possible effects on inflammation and renal outcomes in chronic kidney disease (118). In preclinical models of painful diabetic neuropathy, multi-strain probiotics have been associated with improved blood–nerve barrier markers and reduced peripheral neuroinflammation (38). By contrast, putative gut–brain effects are likely to be mediated indirectly through immune, endocrine, and vagal pathways rather than through direct central nervous system entry, which may partly explain the variable outcomes of human psychobiotic studies (115). Maternal probiotic or synbiotic interventions have also been associated with changes in childhood eczema and asthma-related outcomes, although causal pathways and optimal interventions remain uncertain (14, 115).

The organ-specific molecular pathways through which probiotic-derived signals may influence distant tissues are summarized in Figure 4.

**Figure 4:**
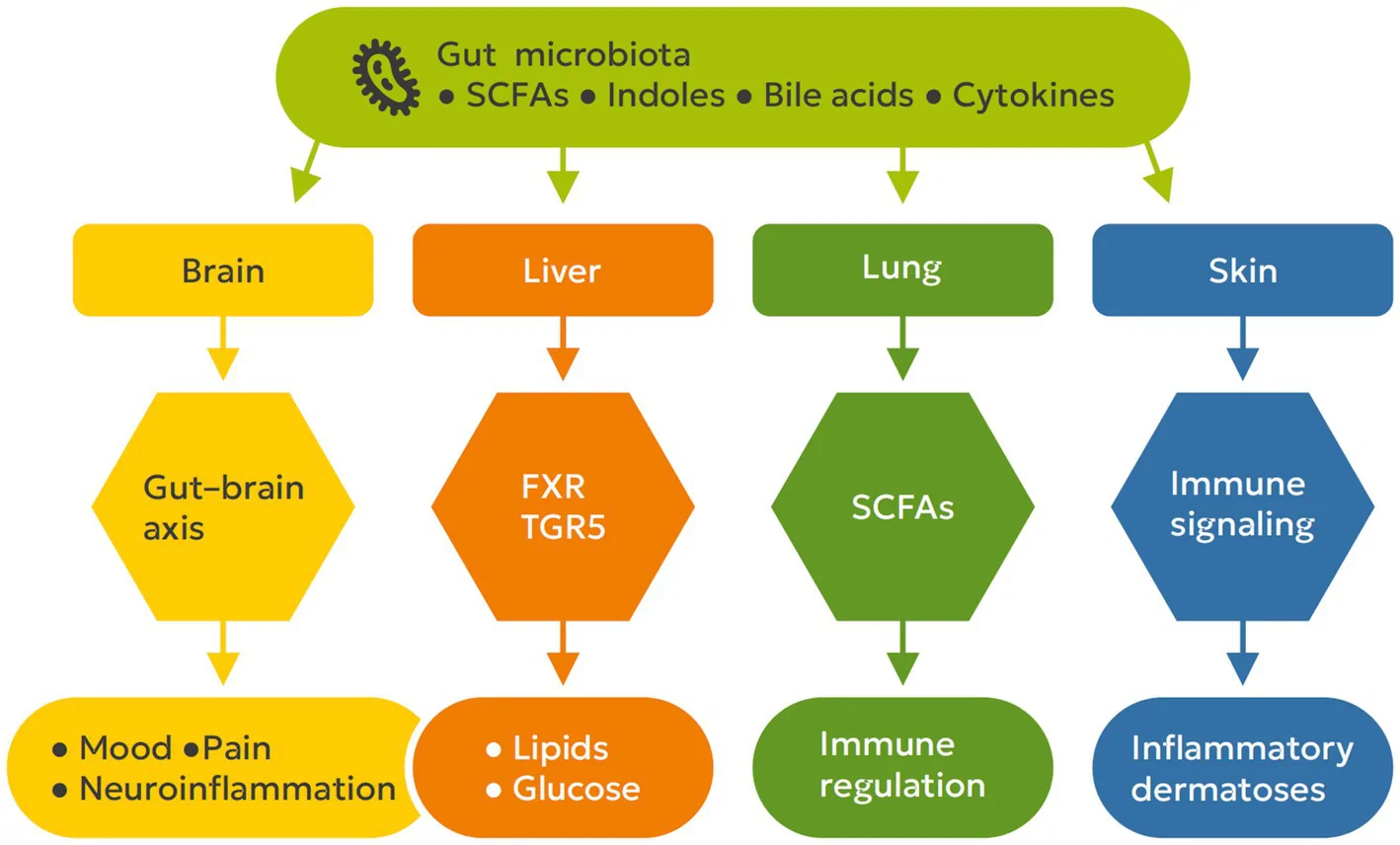
Conceptual overview illustrating how microbiota-derived metabolites and signaling molecules mediate communication between the intestine and distant organs. Microbial products, including short-chain fatty acids, bile acid derivatives, indole metabolites, and immune mediators, contribute to organ-specific regulation of neurological, metabolic, pulmonary, and cutaneous functions through the gut–brain, gut–liver, gut–lung, and gut–skin axes. This figure summarizes the principal molecular pathways underlying the systemic effects of probiotics beyond the gastrointestinal tract.

Taken together, delivery technologies and extra-intestinal applications broaden the range of mechanisms that may be considered in microbiome-based interventions. OMVs and defined postbiotic preparations may be of particular interest when live bacterial administration presents additional safety concerns, including in immunocompromised, neonatal, or oncology settings (113, 119). Encapsulation may also support site-specific co-delivery strategies, for example in approaches intended to modify β-glucuronidase-rich colonic communities and reduce chemotherapy-associated mucosal toxicity (63). Progress will require rigorous comparison of delivery matrices, microbial phenotypes, biodistribution, and clinically meaningful organ-axis outcomes rather than extrapolation from surrogate or preclinical findings alone.

## 11 Conclusion

The evidence reviewed here suggests that probiotic effects arise from interactions among microbial functions, host biology, and the surrounding microbial ecosystem, with outcomes varying by strain and clinical context (31). The nine-block framework presented in this review connects microbial gene functions and metabolic products with host receptors, intracellular signaling pathways, and epithelial or immune responses. It provides a structured way to interpret SCFA production, bile acid and tryptophan metabolism, antimicrobial mechanisms, barrier regulation, immune signaling, engineered microbes, pharmacomicrobiomics, and advanced delivery systems without treating these processes as independent pathways. Clinical translation nevertheless remains constrained by variation in baseline microbiome composition, host physiology, intervention design, and the limited reproducibility of many preclinical findings. More consistent progress will depend on strain-specific characterization, mechanistically informative biomarkers, and well-designed human studies that test clinically meaningful outcomes. This approach may support the rational development and validation of NGPs, LBPs, postbiotic preparations, and related microbiome-based interventions.

## 12 Future perspectives

Translating mechanistic insights into more predictable clinical outcomes will require several coordinated steps. Clinical studies of probiotics, NGPs, and LBPs would benefit from standardized protocols and core outcome sets that permit meaningful comparison across heterogeneous cohorts (31, 120). Biomarkers linked to epithelial integrity, inflammatory activity, or microbial metabolic capacity should be evaluated for their ability to identify likely responders before treatment. Longitudinal metagenomic, metatranscriptomic, and metabolomic data may support patient stratification, provided that candidate signatures are prospectively validated (121). Progress in engineered live therapeutics will also depend on harmonized regulatory expectations and proportionate biosafety requirements (6). Finally, AI and machine-learning models should be tested prospectively for their capacity to predict host–microbiome dynamics and to guide the design of rational synbiotic or microbial interventions, rather than being treated as stand-alone solutions (15).

## 13 Limitations and critical research gaps

Several limitations constrain immediate clinical translation of the nine-block framework. Inter-individual variation in baseline microbiota, geography, diet, and host physiology may contribute to inconsistent colonization, variable study outcomes, and heterogeneous therapeutic responses across populations (15, 31, 120). Much of the available mechanistic evidence is derived from *in vitro* experiments or germ-free and other animal models, and its applicability to human disease remains uncertain (29, 120). Additional challenges include the manufacture and stabilization of oxygen-sensitive NGPs, the validation of biocontainment systems for engineered strains, and the lack of standardized clinical endpoints. Larger, well-powered randomized controlled trials with defined formulations, longitudinal follow-up, and clinically relevant outcome measures are needed to establish effective dosing, safety, and the circumstances in which microbiome-based interventions provide meaningful benefit, particularly in vulnerable populations.
