# Supplementary material for: Biochemical and molecular mechanisms of probiotic action: current concepts and emerging therapeutic applications
Source: Front Nutr. 2026 Oct 7;13:1964473. doi: 10.3389/fnut.2026.1964473 (PMC13684332; doi:10.3389/fnut.2026.1964473)
Supplement: Supplementary file 1 [file Data_Sheet_1.PDF]

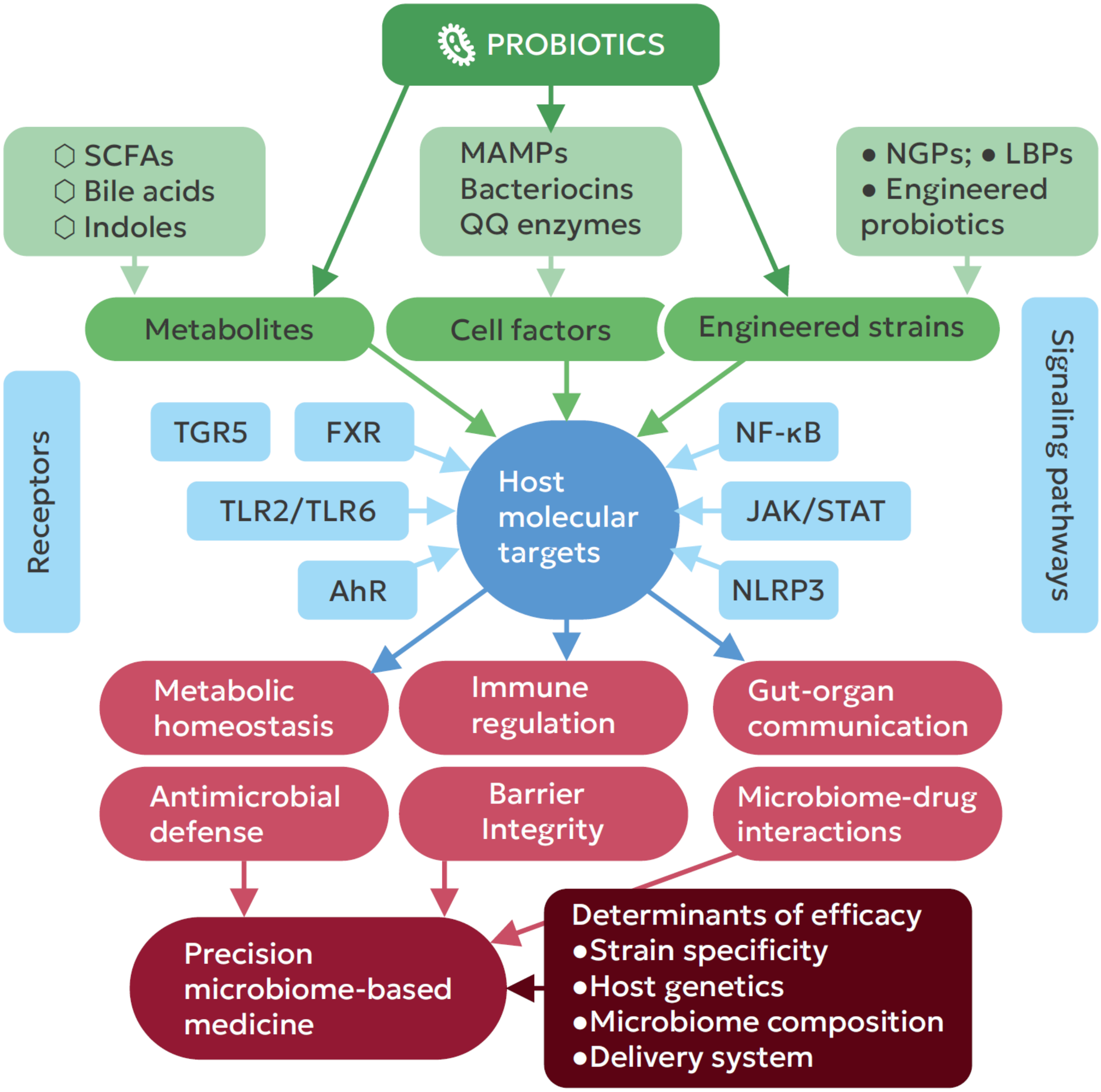

Graphical Abstract. Schematic overview of the principal biochemical and molecular mechanisms underlying probiotic action. Probiotic-derived metabolites, structural cell components, and engineered strains interact with host receptors and intracellular signaling pathways to regulate immune responses, epithelial barrier integrity, metabolic homeostasis, antimicrobial defense, microbiome-drug interactions, and gut-organ communication. These mechanisms collectively provide the foundation for precision microbiome-based medicine.

Abbreviations: SCFAs, short-chain fatty acids; MAMPs, microbe-associated molecular patterns; QQ, quorum quenching; NGPs, next-generation probiotics; LBPs, live biotherapeutic products; FXR, farnesoid X receptor; TGR5, Takeda G protein-coupled receptor 5; AhR, aryl hydrocarbon receptor; TLR2/TLR6, Toll-like receptor 2/6; NF-κB, nuclear factor κB; JAK/STAT, Janus kinase/signal transducer and activator of transcription; NLRP3, NOD-, LRR-, and pyrin domain-containing protein 3 inflammasome.
