# Supplementary material for: Biochemical and molecular mechanisms of probiotic action: current concepts and emerging therapeutic applications
Source: Front Nutr. 2026 Oct 7;13:1964473. doi: 10.3389/fnut.2026.1964473 (PMC13684332; doi:10.3389/fnut.2026.1964473)
Supplement: Supplementary file 2 [file Table_1.DOCX]

**Supplementary Table S1. Taxonomic classification and functional relevance of beneficial, clinically relevant, and mechanistically informative bacteria discussed in the review.**

| **Phylum** | **Family** | **Genus** | **Representative species or strain** | **Functional characteristics** | **Main mechanisms** | **Therapeutic or mechanistic relevance** |
| --- | --- | --- | --- | --- | --- | --- |
| Firmicutes | Lactobacillaceae | *Lactiplantibacillus* | *Lactiplantibacillus plantarum* (formerly *Lactobacillus plantarum*) | Facultative anaerobe; commonly used probiotic | SCFA-related metabolism, barrier-associated effects, immune modulation | Investigated in gastrointestinal and metabolic settings; adjunctive immune modulation |
| Firmicutes | Lactobacillaceae | *Limosilactobacillus* | *Limosilactobacillus reuteri* (formerly *Lactobacillus reuteri*) | Facultative anaerobe; mucosal commensal | Antimicrobial metabolite production, immune modulation, barrier support | Gastrointestinal health; potential metabolic and immune applications |
| Firmicutes | Lactobacillaceae | *Lacticaseibacillus* | *Lacticaseibacillus rhamnosus* (formerly *Lactobacillus rhamnosus*) | Facultative anaerobe; well-studied probiotic | Barrier reinforcement, immune modulation, pathogen inhibition | Prevention of antibiotic-associated diarrhea; support of intestinal barrier function |
| Actinobacteriota | Bifidobacteriaceae | *Bifidobacterium* | *Bifidobacterium longum* | Anaerobic commensal; prevalent in infant and adult gut communities | SCFA production, immune modulation, competitive effects on pathogens | Metabolic and immune support; potential relevance to gut–brain communication |
| Actinobacteriota | Bifidobacteriaceae | *Bifidobacterium* | *Bifidobacterium longum* subsp. *infantis* | Anaerobic commensal; prominent in breast-fed infants | Carbohydrate utilization, SCFA production, mucosal immune modulation | Early-life microbiome development and immune maturation |
| Actinobacteriota | Bifidobacteriaceae | *Bifidobacterium* | *Bifidobacterium breve* | Anaerobic commensal; infant-associated species | SCFA production and immune modulation | Infant gut health; potential metabolic and immune applications |
| Actinobacteriota | Bifidobacteriaceae | *Bifidobacterium* | *Bifidobacterium bifidum* BB1 | Anaerobic commensal; strain-specific epithelial effects | Selective enhancement of occludin expression and junctional localization through TLR2/TLR6-associated signaling | Support of intestinal barrier integrity; potential adjunctive relevance in barrier-related disorders |
| Proteobacteria | Enterobacteriaceae | *Escherichia* | *Escherichia coli* Nissle 1917 | Commensal strain; frequently used engineered microbial chassis | Competitive exclusion; engineered delivery of anti-inflammatory mediators or STING agonists | Inflammatory bowel disease research, tumor-targeted immunotherapy, and engineered live biotherapeutic platforms |
| Firmicutes | Lactococcaceae | *Lactococcus* | *Lactococcus lactis* | Facultative anaerobe; food-grade organism | Experimental expression and delivery of anti-inflammatory mediators, including IL-10 | Experimental colitis and engineered microbial platforms |
| Firmicutes | Leuconostocaceae | *Leuconostoc* | *Leuconostoc lactis* EJ-1* | Facultative anaerobe; strain investigated in experimental colitis | NF-κB-associated inflammatory modulation and macrophage polarization | Experimental colitis and immunomodulation |
| Firmicutes | Enterococcaceae | *Enterococcus* | *Enterococcus* spp. | Facultative anaerobes; components of selected probiotic cocktails | Ecological competition and metabolic contributions within multi-strain formulations | Adjunctive use in selected probiotic formulations |
| Firmicutes | — | Butyrate-producing anaerobes/consortia | Multiple genera, including *Faecalibacterium* and *Roseburia* spp. | Anaerobic fermentation of dietary fibres | Butyrate production, HDAC-associated signaling, GPR41/GPR43 signaling, and Treg-related effects | Inflammatory and metabolic disorders; barrier reinforcement |
| — | — | BSH-active probiotic strains | Multiple probiotic genera, including lactobacilli and bifidobacteria | Bile salt hydrolase activity | Bile acid deconjugation; effects on FXR/TGR5-associated signaling and cholesterol metabolism | Metabolic syndrome, lipid homeostasis, and cardiovascular-risk-related investigation |
| Actinobacteriota | Eggerthellaceae | *Eggerthella* | *Eggerthella lenta* | Anaerobic commensal | Digoxin reduction through the *cgr* operon | Pharmacomicrobiomic model of variable drug efficacy |
| Firmicutes | Enterococcaceae | *Enterococcus* | *Enterococcus faecalis* | Facultative anaerobe; opportunistic pathogen | Altered membrane properties associated with reduced susceptibility to cationic antimicrobial peptides | Model organism for antimicrobial resistance and gut-pathogen dynamics |
| Proteobacteria | Pseudomonadaceae | *Pseudomonas* | *Pseudomonas aeruginosa* | Opportunistic pathogen | Acyl-homoserine-lactone-mediated quorum sensing, biofilm formation, and virulence regulation | Experimental model for quorum-quenching and antivirulence strategies |
| Proteobacteria | Enterobacteriaceae | *Erwinia* | *Erwinia carotovora* | Plant pathogen | Acyl-homoserine-lactone-dependent quorum sensing | Experimental model for AiiA-mediated quorum-quenching studies |

*The designation *Leuconostoc lactis* EJ-1 is retained as reported in the cited experimental study; its current taxonomic status should be checked only if the authors choose to update the underlying source nomenclature.

**Notes to Supplementary Table S1**

- Current genus names are used for reclassified former *Lactobacillus* species where possible. Former names are retained in parentheses to facilitate comparison with legacy clinical and commercial literature.
- *Bifidobacterium longum* subsp. *infantis* is used in place of the commonly used legacy designation *Bifidobacterium infantis*.
- Functional groups, including butyrate-producing anaerobes and BSH-active probiotic strains, are presented without assigning all member taxa to individual species because they are discussed in the main text primarily as functional communities.
- *Eggerthella lenta*, *Enterococcus faecalis*, *Pseudomonas aeruginosa*, and *Erwinia carotovora* are included as mechanistically informative organisms rather than beneficial probiotics. They are discussed in relation to pharmacomicrobiomics, antimicrobial peptide resistance, and quorum-quenching models.
