# Supplementary material for: Biochemical and molecular mechanisms of probiotic action: current concepts and emerging therapeutic applications
Source: Front Nutr. 2026 Oct 7;13:1964473. doi: 10.3389/fnut.2026.1964473 (PMC13684332; doi:10.3389/fnut.2026.1964473)
Supplement: Supplementary file 3 [file Table_2.DOCX]

**Supplementary Table S2.** Major bioactive microbial products, metabolites, and their principal molecular targets.

| Microbial product or metabolite | Produced by | Host receptor or primary molecular target | Molecular pathway | Biological effect | Representative examples and key references |
| --- | --- | --- | --- | --- | --- |
| Butyrate | Fiber-fermenting gut commensals and butyrate-producing anaerobes | GPR41, GPR43, HDACs, colonocyte metabolic pathways | SCFA signaling, epigenetic regulation, immune modulation | Colonocyte energy supply, anti-inflammatory activity, barrier reinforcement | Butyrate as a central SCFA-derived postbiotic mediator (17,18,21,25) |
| Propionate | Microbial fermentation of dietary fibers and other fermentable substrates | GPR41, GPR43, intestinal and hepatic metabolic pathways | SCFA signaling, glucose and lipid regulatory pathways | Metabolic regulation, enteroendocrine signaling, immunometabolic effects | Propionate within the SCFA signaling network (17,19,25) |
| Acetate | Broad gut microbial carbohydrate fermentation | GPR41, GPR43, peripheral metabolic tissues | SCFA signaling and microbial cross-feeding pathways | Systemic metabolic signaling, energy homeostasis, substrate support for downstream metabolite production | Acetate as a major microbiota-derived fermentation product (16,17,25) |
| Secondary bile acids | Gut microbes expressing bile salt hydrolase and related bile acid-transforming enzymes | FXR, TGR5 | Bile acid receptor signaling and endocrine-metabolic regulation | Lipid homeostasis, GLP-1-related effects, gut-liver axis signaling | Microbially modified bile acids as host signaling ligands (27-30) |
| Indole-3-aldehyde | Microbial tryptophan metabolism | AhR | AhR-dependent mucosal immune signaling | Mucosal homeostasis, immune tolerance, barrier support | Tryptophan-derived indole signaling in host–microbe communication (37,38,45) |
| Lactate | Probiotic and commensal bacteria during anaerobic glycolysis and fermentation | Epithelial metabolic pathways and microbial cross-feeding networks | Lactate-shuttle and fermentation-linked metabolic signaling | Support of cross-feeding, precursor role in SCFA production, epithelial metabolic support | Lactate as an intermediate in probiotic metabolic networks (16,23,25) |
| Bacteriocins | Probiotic bacteria, especially lactic acid bacteria | Bacterial membranes and pathogen-associated molecular targets | Direct antimicrobial peptide action | Pathogen inhibition, reduced colonization, suppression of competing microbes | Pediocin, MccJ25, and related antimicrobial peptides (72,74,78) |
| Organic acids | Lactic acid bacteria and other fermentative commensals | Microbial membranes and pH-sensitive pathogen-associated systems | Luminal acidification and membrane-disruptive antimicrobial action | Pathogen growth inhibition, ecological competition, antimicrobial support | Lactic acid and related antimicrobial organic acids (16,72,73) |
| EPS | Exopolysaccharide-producing probiotic strains | Epithelial and immune cell interface, including pattern-recognition-related pathways | Host–microbe interface modulation and biofilm-associated signaling | Barrier support, immune modulation, mucosal protection | Probiotic surface and secreted structures involved in host signaling (22,53,61) |
| OMVs | Gram-negative commensals and engineered microbial systems | TLRs, epithelial cells, antigen-presenting cells | Vesicle-mediated cargo delivery and immune signaling | Postbiotic immunomodulation, targeted host signaling, non-living delivery potential | OMV-based postbiotic and delivery concepts (14,94,120) |

This table summarizes the principal microbial metabolites and bioactive products discussed across the review, together with their major host receptors or primary molecular targets, associated signaling pathways, and biological effects. It integrates metabolic, immune, antimicrobial, and delivery-related mechanisms into a single mechanistic framework.
